# Crystal structure of *Methanococcus jannaschii* dihydroorotase with substrate bound

**DOI:** 10.1107/S2053230X25010556

**Published:** 2026-01-01

**Authors:** Jacqueline Vitali, Jay C. Nix, Haley E. Newman, Michael J. Colaneri

**Affiliations:** a Department of Physics Cleveland State University Cleveland Ohio USA; b Department of Biological, Geological and Environmental Sciences Cleveland State University Cleveland Ohio USA; c Molecular Biology Consortium, Advanced Light Source Lawrence Berkeley National Laboratory Berkeley California USA; d Department of Chemistry and Physics State University of New York at Old Westbury Old Westbury New York USA; The Scripps Research Institute, USA

**Keywords:** dihydroorotase, *Methanococcus jannaschii*, pyrimidine biosynthesis, flexible loop

## Abstract

Here, we report the X-ray structural analysis of dihydroorotase from *Methanococcus jannaschii* co-crystallized with dihydroorotate at pH 6.5. The crystals are isomorphous with the crystals of the apoenzyme, with space group *P*3_2_21 and unit-cell dimensions *a* = *b* = 111.4, *c* = 101.2 Å. The structure was refined to *R* = 0.169 and *R*_free_ = 0.186 at a resolution of 1.87 Å at room temperature. During crystallization the degradative reaction took place, and the electron density in the active site corresponds to the substrate carbamoyl aspartate. Carbamoyl aspartate interacts with the protein in the active site in a manner similar to that observed for *Escherichia coli* and human dihydroorotases. However, the flexible loop (residues 140–151) adopts both conformations in the crystal, loop-out and loop-in, with the loop-out conformation having higher occupancy. This contrasts with our expectations for the flexible loop to be exclusively in the loop-in conformation, which is the conformation that it adopts to stabilize the binding of the substrate in the known systems. Additional studies with substrate analogs that resemble carbamoyl aspartate and different crystallization conditions would provide further insight into the conformation of the flexible loop in this system.

## Abstract 2

We present the 1.87 Å resolution structure of *M. jannaschii* dihydroorotase complexed with the substrate carbamoyl aspartate at room temperature. Contrary to expectations, a flexible loop which is important in catalysis and closes upon substrate binding in order to stabilize the substrate and transition state is observed in both the loop-out and loop-in conformations, with the loop-out conformation having higher occupancy.

## 1. Introduction

Dihydroorotase (DHOase) is the third enzyme of *de novo* pyrimidine biosynthesis, the pathway that leads to the formation of pyrimidine nucleotides that are the building blocks of DNA and RNA. It catalyzes the reversible cyclization of *N*-carbamoyl-l-aspartate (CA) to form l-dihydro­orotate (DHO; Cooper & Wilson, 1954 ▸; Lieberman & Kornberg, 1954 ▸). DHOases are zinc metalloenzymes and belong to the amidohydrolase superfamily of proteins (Holm & Sander, 1997 ▸). The core domain of all DHOases has the (βα)_8_-barrel fold (TIM barrel; Holm & Sander, 1997 ▸). Dihydroorotases are very diverse (Fields *et al.*, 1999 ▸; Grande-García *et al.*, 2014 ▸), even though they are present in all organisms and have the same core domain and catalytic function.

They have been subdivided among species into types and subtypes (Fields *et al.*, 1999 ▸; Grande-García *et al.*, 2014 ▸), each with different structural characteristics. There are two major types, with very low sequence identity between them (<20%; Fields *et al.*, 1999 ▸). Long DHOases (type I) are more ancient and larger, with a molecular weight of ∼45 kDa. Short DHOases (type II) are more recent, with a molecular weight of ∼38 kDa. According to a recent phylogenetic analysis (Grande-García *et al.*, 2014 ▸), long DHOases subdivide into archaeal, bacterial type I, bacterial type III, human CAD and inactive CAD in fungi. Short DHOases correspond to bacterial type II, plant and active DHOases in fungi. Each subtype has specific structural characteristics (Grande-García *et al.*, 2014 ▸; Lipowska *et al.*, 2019 ▸; Vitali *et al.*, 2023 ▸; Guan *et al.*, 2021*a* ▸). The only subtype without a representative structure is that of plant DHOases. In addition, some form different quaternary associations with themselves, unique to each subtype. For example, both bacterial types I and II form dimers and the mode of dimerization seems to differ in the two subtypes (Grande-García *et al.*, 2014 ▸). Also, human DHOases form dimers in the crystal (Grande-García *et al.*, 2014 ▸). *Saccharomyces cerevisiae* DHOase (ScDHOase) forms tetramers in the crystalline state and dimers and tetramers in solution (Guan *et al.*, 2021*a* ▸). Some DHOases form non­covalent complexes with ATCase, as is the case for *Aquifex aeolicus* DHOase (AaDHOase; Zhang *et al.*, 2009 ▸). Finally, in mammals, the three enzymes of the pathway, ATCase, CPSase and DHOase, are part of the same polypeptide chain and form hexamers (Evans & Guy, 2004 ▸; Moreno-Morcillo *et al.*, 2017 ▸).

The active site is highly conserved among DHOases. Typically, it contains two zinc ions that are coordinated by invariant residues: four His and one Asp (Thoden *et al.*, 2001 ▸; Grande-García *et al.*, 2014 ▸). In addition, all types, except for bacterial type I, contain a carboxylated Lys that also bridges the two zinc ions. Bacterial type I uses an Asp, invariant in that subtype, to bridge the two zinc ions (Mehboob *et al.*, 2010 ▸). Furthermore, studies with *Escherichia coli* DHOase (EcDHOase; Thoden *et al.*, 2001 ▸; Lee *et al.*, 2005 ▸) and human DHOase (huDHOase; Grande-García *et al.*, 2014 ▸) showed that the residues and positions in the active site that bind to the substrate and product are highly conserved and rigid among dihydrorotases. The only flexibility in the active site is in a long flexible loop that closes as a lid during catalysis (loop-in form), stabilizing the substrate and the transition state (Lee *et al.*, 2005 ▸; del Caño-Ochoa *et al.*, 2018 ▸), with a two-residue signature for binding the substrate that is unique to each type. In the apo form and in the presence of the product DHO, this loop is in an open conformation (loop-out), exposing the active site and facilitating product release during catalysis. As an exception, bacterial type I has a short flexible loop that interacts minimally with the substrate (Rice *et al.*, 2016 ▸; Mehboob *et al.*, 2010 ▸), but it appears that these DHOases are poised to interact with ATCase (Grande-García *et al.*, 2014 ▸), as is the case in AaDHOase (Zhang *et al.*, 2009 ▸) and possibly *Bacillus anthracis* DHOase (Kankanala, 2011 ▸). Deviations from these regularities are seen in some species (Guan *et al.*, 2021*a* ▸,*b* ▸,*c* ▸,*d* ▸; Lee, Chan *et al.*, 2007 ▸; Lin *et al.*, 2023 ▸; Huang, 2025 ▸) with ligands, but have not been observed with the natural substrate and product.

The DHOase from *Methanococcus jannaschii* (MjDHOase), a hyperthermophilic and barophilic archaeon (Jones *et al.*, 1983 ▸), is the only archaeal DHOase that has been studied. We have previously carried out a kinetic characterization (Vitali *et al.*, 2017 ▸; Ayotte & Vitali, 2022 ▸). Its specific activity increases with temperature in both biosynthetic and degradative directions and, at saturation, the specific activity of the degradative reaction was almost twice that of the biosynthetic reaction at all temperatures studied. The DHOase reaction is pH-dependent (Ayotte & Vitali, 2022 ▸). The pH profile of MjDHOase was similar to other DHOases. At low pH (∼5.5) the biosynthetic reaction is favored and at high pH (∼6.5–9) the degradative reaction is favored (Ayotte & Vitali, 2022 ▸). We previously determined the apo structure of this enzyme (Vitali *et al.*, 2023 ▸), providing the structural features of archaeal DHOases and their similarities to and differences from other types of DHOase. In the present study, we crystallized the enzyme in the presence of DHO in order to understand how the substrate and/or product bind and compare this with other DHOases. Given the reversibility of the reaction, we expected the crystals to contain DHO and CA to different extents, similar to huDHOase (Grande-García *et al.*, 2014 ▸). Furthermore, we expected the flexible loop to be in the loop-in conformation when CA is bound and in the loop-out conformation when DHO is bound. It turned out that the ligand in the active site was CA, which is consistent with the degradative reaction being predominant at the pH of crystallization (pH 6.5). However, unexpectedly, we found the flexible loop in both the loop-out and loop-in conformations, with the loop-out conformation being predominant. This suggests either a mixed population of protein molecules in the crystal or a dynamic equilibrium between the two conformations in the protein, resulting in an averaged electron density representing both conformations.

## 2. Materials and methods

### 2.1. Protein preparation and crystallization

Purification was carried out as described previously (Vitali *et al.*, 2017 ▸) from Rosetta-gami2 (DE3) cells harboring the pET-21a plasmid with the *M. jannaschii pyrC* gene. It involved an ammonium sulfate precipitation step, a heat step at 85°C for 15 min and chromatography using an SP cation-exchange column and a Phenyl Sepharose column. For the crystal used in this study, the protein was at a concentration of 6.7 mg ml^−1^ and the protein buffer was 50 m*M* Tris–HCl pH 7.5, 2 m*M* β-mercaptoethanol, 0.05 m*M* zinc acetate. The reservoirs contained 35% PEG 400, 0.1 *M* zinc acetate, 0.1 *M* Na MES pH 6.5. The drops consisted of 2 µl protein solution, 1 µl reservoir solution and 0.5 µl acqueous DHO stock at 65 m*M*.

### 2.2. Data collection and processing

The crystal was mounted in a 0.8 mm quartz capillary by conventional means (McPherson, 1989 ▸). X-ray data were measured on Advanced Light Source (ALS) beamline 4.2.2 at a temperature of ∼298 K. The crystal-to-detector distance was 200 mm and the wavelength used was 1.0 Å. Images were processed with *XDS* (Kabsch, 2010 ▸). Structure-factor amplitudes were obtained by scaling and merging intensities using *AIMLESS* (Evans & Murshudov, 2013 ▸). Processing statistics are shown in Table 1 ▸.

### 2.3. Structure solution and refinement

Structure solution and refinement were carried out using *Phenix* (Liebschner *et al.*, 2019 ▸). The initial model consisted of the apo structure without residues 141–149 of the flexible loop and water molecules, and was positioned in the unit cell with *Phaser* in *Phenix*. Refinement was carried out using *phenix.refine*. Model building was performed using *Coot* (Emsley & Cowtan, 2004 ▸). 2*mF*_o_ − *DF*_c_ and *mF*_o_ − *DF*_c_ electron-density difference maps suggested two alternate conformations for the flexible loop: loop-out and loop-in. The electron density of the loop-out conformation was stronger and similar to that in the apo form (Vitali *et al.*, 2023 ▸). The electron density corresponding to the loop-in form was weaker but was sufficiently convincing for it to be modelled and included in the model. The electron density of the active site contained only the substrate CA. The structure contains 11 Zn ions: two catalytic ions in the active site and nine interstitial ions as in the apo structure. The group occupancies of the two alternate conformations of the flexible loop, the ligand and the zinc ions were included in the refinement. The positions of the zinc ions were confirmed from an anomalous difference map at the end of the analysis.

## 3. Results and discussion

### 3.1. Structural aspects and the active site

The model presented in this paper consists of one asymmetric unit of 3449 non-H protein atoms in 423 amino acids (including a carboxylated Lys and two conformations of residues 141–149 of the flexible loop), 12 atoms of the ligand carbamoyl aspartate (CA), two zinc ions in the active site and nine interstitial zinc ions, and 186 water molecules. The biological unit is a monomer, as discussed for the apo structure (Vitali *et al.*, 2023 ▸). A summary of the model-refinement statistics is given in Table 2 ▸. The Ramachandran plot showed that 95.69% of the residues lie in the most favored regions and only four residues are outliers, with φ and ψ angles of 68.3° and −82.1° for Asp11, −166.2° and 36.5° for Asn12, 23.2° and 68.5° for His227 and −157.3° and −1.5° for Gly324. The electron density for His227 and Gly324 is well defined, but is poorly defined for Asp11 and Asn12.

The electron density in the active site corresponded to CA, as there was no electron density for the N3 atom and its associated bonds in a DHO configuration. During crystallization at pH 6.5, the DHO in solution was converted to CA by the enzyme because at this pH the activity of the degradative reaction is significantly greater than that of the biosynthetic reaction (Ayotte & Vitali, 2022 ▸). Of the two alternate conformations of the flexible loop, loop-out and loop-in, the loop-out conformation had strong well defined electron density and was similar to the apo form (Vitali *et al.*, 2023 ▸). The loop-in conformation corresponded to weaker electron density and there was not an independent experimental confirmation for it from another structure. The occupancy factors were 0.6 for the loop-out conformation and 0.4 for the loop-in conformation. The real-space correlation coefficients (RSCC) of residues Val141, Lys142, Gly145, Asp146, Leu147 and Phe148 were high, mostly >0.9, and were comparable in the two conformations. The RSCCs for Ser143 and Val144 were lower for the loop-in form, 0.85 and 0.83, respectively, compared with the loop-out form, 0.91 and 0.96, respectively. Yet, the electron density was sufficiently convincing to include them in the model. Typically, values of RSCC between 0.8 and 0.9 correspond to less well modeled parts which nonetheless exist (Deller & Rupp, 2015 ▸).

However, after the analysis was completed, we determined the flexible-loop conformation in MjDHOase computationally for the isolated enzyme, as well as in the presence of citrate, using the *AlphaFold*3 online server (Abramson *et al.*, 2024 ▸) for prediction of the 3D structures of biomolecular complexes, including proteins and ligands. The server only includes certain ligands, and CA was not one of them. However, citrate, which is a close analog of CA, was included. In addition, carboxylated lysine (KCX) was not one of the modified residues included in the server and was not included in the computation. Interestingly, the predicted flexible loop for the isolated enzyme was in the loop-out conformation and was closely similar to that determined crystallographically for the wild-type enzyme. The root-mean-square deviation (r.m.s.d.) between the C^α^ atoms of the two structures was 0.4 Å (Vitali *et al.*, 2023 ▸). For the complex, the computed citrate was near the CA in the present structure but was not superimposable. Furthermore, the predicted conformation of the flexible loop in the presence of citrate was similar to the loop-in conformation that we fitted in the electron density of the present structure, with an r.m.s.d. of 0.4 Å between the C^α^ atoms of the two structures, providing support for the crystallographically determined loop-in conformation.

An inspection of the final 2*mF*_o_ − *DF*_c_ map, contoured at 1.0σ, shows continuous electron density for all main-chain and side-chain atoms, with a few exceptions. There are gaps in the electron density between residues Lys10 and Asp11 and between residues Lys142 and Ser143 of the loop-in conformation, between the main-chain and side-chain atoms of Val143 of the loop-in conformation, there is no electron density for the side chains of a few surface groups, mostly Lys and Glu, and the last two residues are poorly defined.

**Figure 1:**
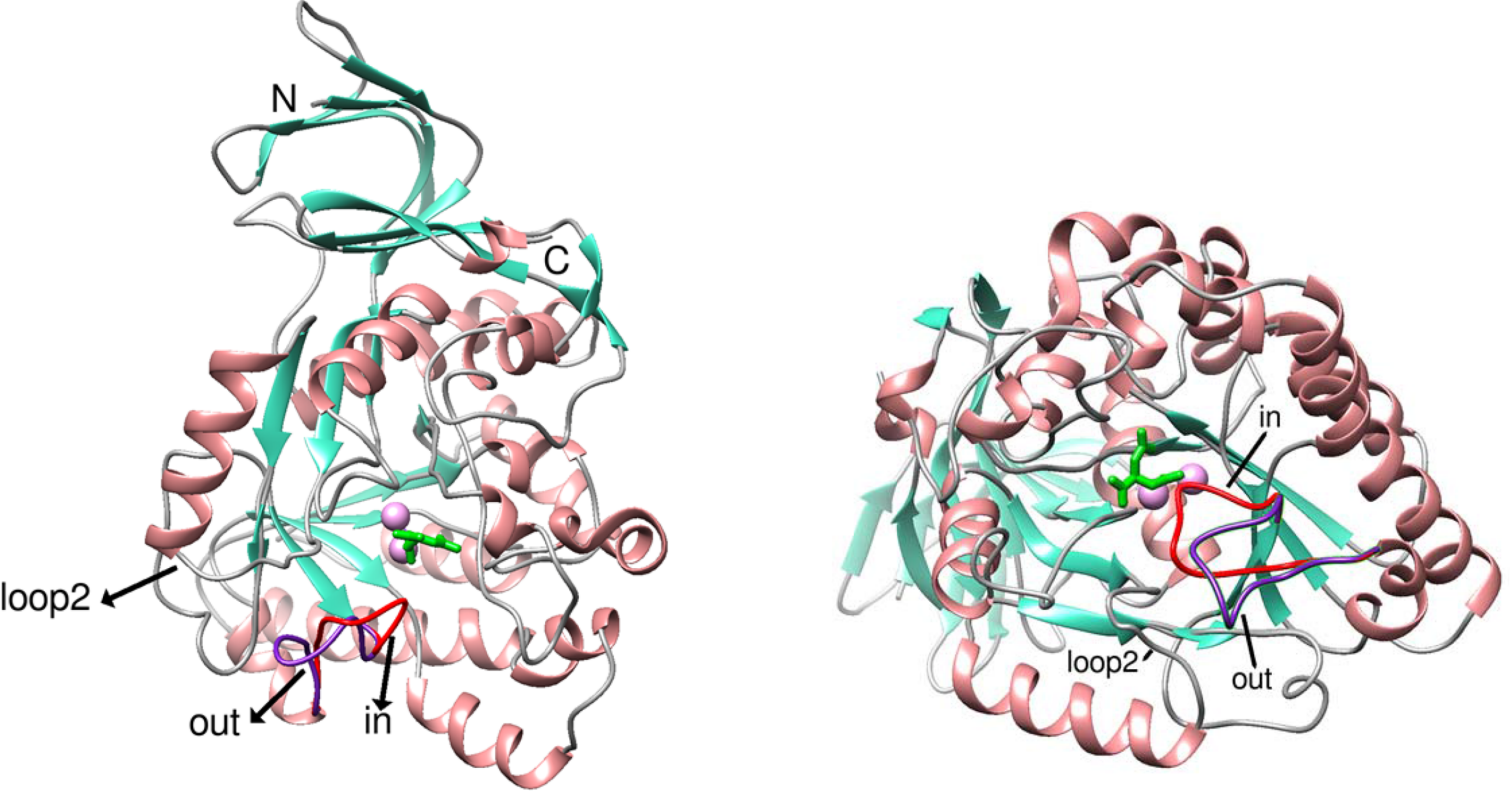
Cartoon diagrams of MjDHOase with the flexible loop in the two alternate conformations indicated. Helices are depicted in salmon, strands in teal and loops in gray, except for the flexible loop, which is shown in red for the loop-in conformation and in purple for the loop-out conformation and its two conformations are indicated. Loop 2 that makes contacts with the flexible loop in the loop-out conformation is also indicated. The two zinc ions and the substrate CA in the active site are included. The zinc ions are shown as plum-colored spheres and the substrate CA is in lime green. The two views are nearly perpendicular to each other.

**Figure 2:**
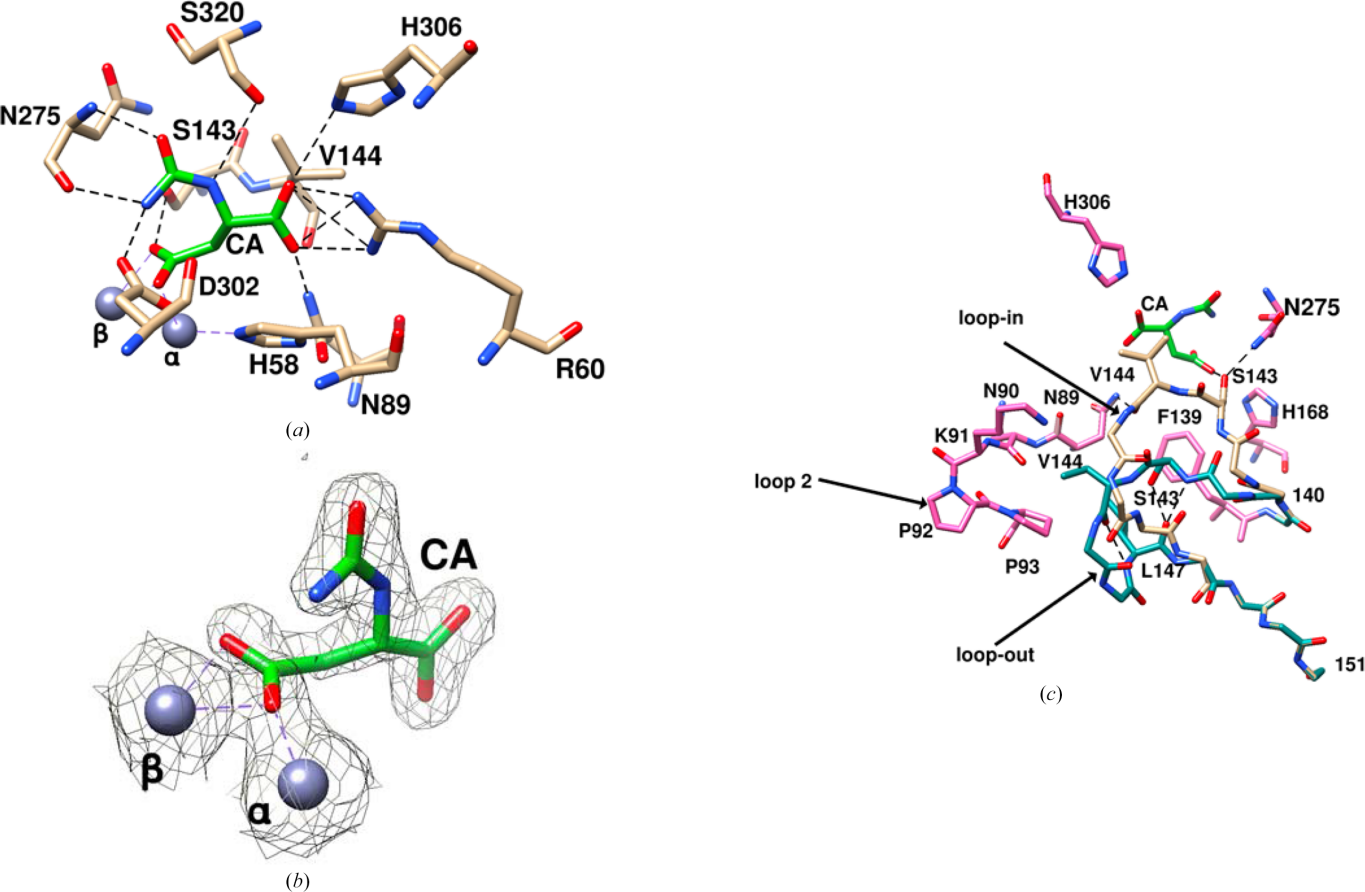
Interactions of CA in the active site. The C^α^ atoms of CA are in lime green and those of the protein are in tan. The two zinc ions are in gray. (*b*) The electron density around CA and the two zinc ions in gray corresponds to the 2*mF*_o_ − *DF*_c_ map, and the contour level is 0.9σ. The electron density confirms that the ligand is CA. (*c*) Interactions of Ser143 and Val144 with the protein in both loop-in and loop-out conformations. The two conformations of the flexible loop are shown in tan for the loop-in conformation and in dark cyan for the loop-out conformation. The loops are exhibited as polyalanine chains, except for Ser143 and Val144 for the loop-in conformation and Ser143, Val144 and Leu147 for the loop-out conformation. The protein residues that interact with the loop atoms are shown in hot pink unless they are also part of the loop. The substrate CA is shown in lime green. Hydrogen bonds are shown by dashed lines. Two hydrogen bonds to the protein (Asn89 ND2⋯Val144 O and Asn275 ND2⋯Ser143 OG) and one to CA (Ser143 OG⋯CA424 O5) are made for the loop-in conformation and contacts with His168, His306 and the α-carboxylate of CA (see Supplementary Tables S2 and S3). Three intraloop hydrogen bonds are made in the loop-out conformation (Ser143 OG⋯Leu147 O, Ser143 N⋯Leu147 O and Leu147 N⋯Val144 O) and contacts with Phe139 and Leu147, and with Asn89, Lys91 and Pro93 of loop 2 (see Supplementary Table S3).

A view of the protein including the two alternate conformations of the flexible loop, the bound CA and the two zinc ions in the active site is shown in Fig. 1 ▸ and Supplementary Fig. S1. The structure is very similar to that of the apoenzyme, with an r.m.s.d. between corresponding C^α^ atoms of 0.1 Å using the loop-out conformation of the flexible loop. The primary difference is the second conformation of the flexible loop, with the apoenzyme being in the loop-out conformation and the complex having two conformations of the flexible loop, loop-out and loop-in, even though the loop-out conformation has a higher occupancy. The distance between the C^α^ atoms of Ser143 in the two conformations is 4.16 Å and that between the OG atoms of the same residue is 7.15 Å. The 2*mF*_o_ − *DF*_c_ map at the site of the ligand is included in Fig. 2 ▸(*b*). The 2*mF*_o_ − *DF*_c_ maps for the loop-in and loop-out conformations of the flexible loop are shown in Supplementary Fig. S2.

The interactions of CA with the enzyme are shown in Fig. 2 ▸(*a*). Hydrogen bonds and salt bridges are summarized in Supplementary Table S1. As shown in Fig. 2 ▸(*a*) and Supplementary Table S1, the α-carboxylate (O62–C61–O61) interacts with the side chains of Arg60, His306 and Asn89, the N1 atom interacts with the main-chain carbonyl of Ser320, the carbamoyl O2 and N3 atoms interact with the main-chain N and O atoms of Asn275, and the carbamoyl N3 atom also interacts with the side chain of the invariant Asp302. The β-carboxylate of CA is coordinated to the zinc ions. In addition, the β-carboxylate of CA hydrogen-bonds to OG of Ser143 in the loop-in conformation of the flexible loop and the α-carboxylate O61–C61–O62 makes van der Waals contacts with Val144 of the same conformation, with the shortest distance being O62⋯CG2 at 3.24 Å. On the other side, the α-carboxylate makes an edge-on vertical interaction with His58, with the shortest distance being O61⋯ND1 at 3.28 Å. Arg60, His306, Asn89, His58 and Asp302 are invariant among DHOases (Thoden *et al.*, 2001 ▸; Vitali *et al.*, 2017 ▸), while Ser320 and Asn275, which use their main chains to interact with CA, correspond to invariant positions.

In addition to CA, Ser143 and Val144 of the flexible loop are also involved in two hydrogen bonds to nearby protein residues: Asn275 ND2⋯Ser143 OG and Asn89 ND2⋯Val144 O. Ser143 and Val144 also make contacts with His168 and His306, respectively. These interactions are included in Fig. 2 ▸(*c*) and are listed in Supplementary Tables S2 and S3.

The same two residues, in the loop-out conformation, are involved in three intraloop hydrogen bonds: Ser143 OG⋯Leu147 O, Ser143 N⋯Leu147 O and Leu147 N⋯Val144 O. In addition, Ser143 makes contacts with Phe139 and Leu147, and Val144 makes contacts with Asn89, Lys91 and Pro93 on the surface of the protein. These interactions are shown in Fig. 2 ▸(*c*) and are listed in Supplementary Table S3.

### 3.2. Comparison of the active site with other DHOases

**Figure 3:**
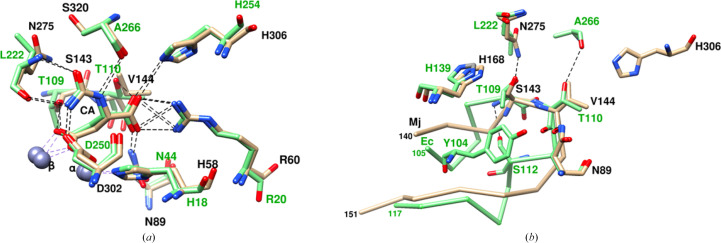
Comparison of the interactions in the active site between MjDHOase and EcDHOase (PDB entry 1xge; Lee *et al.*, 2005 ▸). MjDHOase is in tan and EcDHOase is in light green. The coloring of the labels is black for MjDHOase and green for EcDHOase. (*a*) Only interactions involving CA are shown for clarity. CA is at the center. The figure illustrates the high similarity of the active site in MjDHOase and EcDHOase. (*b*) Interactions of the two residues at the tip of the flexible loop with the protein residues nearby, illustrating the correspondence of some of the interactions and the larger number of interactions in EcDHOase compared with MjDHOase (see also Supplementary Table S2). Hydrogen bonds are indicated by dashed lines. Protein residues that make also contacts of ≤4.0 Å are indicated. The C^α^ backbones of the two flexible loops are included. Thr109 and Thr110 in EcDHOase form three hydrogen bonds (Thr109 N⋯Ser112 OG, The110 OG1⋯Ala266 O and Asn44 ND2⋯Thr110 O), and Thr109 makes contacts with Tyr104, Leu222 and His139. The corresponding Ser143 and Val144 in MjDHOase form two hydrogen bonds (Asn89 ND2⋯Val144 O and Asn275 ND2⋯Ser143 OG) and contacts with His168 and His306, respectively.

The interactions of CA with the protein are similar to those in both EcDHOase (PDB entry 1xge; Lee *et al.*, 2005 ▸) and huDHOase (PDB entry 4c6f; Grande-García *et al.*, 2014 ▸) (Figs. 3 ▸*a* and 4 ▸*a*). These are the only known DHOase structures that have substrate bound. The catalytic elements occupy similar positions in all, whether free or bound to the substrate. The largest differences are in the interactions of the flexible loops with CA, with each species having its own characteristic flexible loop. It is considered that the flexible loop has a key functional role in catalysis and contributes to the differences in activity among DHOases (Lee, Maher, Christopherson *et al.*, 2007 ▸; del Caño-Ochoa *et al.*, 2018 ▸). It shows high variability in size, sequence and structure among DHOases, with features that are unique to each system. Each flexible loop has a two-amino-acid signature with which it interacts with the substrate CA during catalysis, and the interaction with the substrate CA is also considered to be necessary for the loop-in conformation.

In EcDHOase, which is a dimer, one of the subunits (PDB entry 1xge; Lee *et al.*, 2005 ▸) has CA in its active site and the loop-in conformation for the flexible loop. Residues Thr109 and Thr110 at the tip of the flexible loop (EcDHOase residues 105–117) hydrogen-bond to the β- and α-carboxyl groups of CA, respectively (Fig. 3 ▸*a*), stabilizing the loop-in conformation. It was considered that the hydrogen bonds of Thr109 and Thr110 to CA were essential for the stability of the loop-in conformation, as mutations that disrupted these hydrogen bonds showed no electron density for the flexible loop and this was correlated with reduced or diminished activity of the mutant enzymes (Lee, Maher, Christopherson *et al.*, 2007 ▸). These two residues make three additional hydrogen bonds (Fig. 3 ▸*b*, Supplementary Table S2), of which one is intraloop, Thr109 N⋯Ser112 OG, and two with protein residues nearby, Thr110 OG1⋯Ala266 O and Asn44 ND2⋯Thr110 O. Thr109 also makes contacts with Leu222, His139 and Tyr104 (Fig. 3 ▸*b*, Supplementary Table S2). These interactions provide further stability to the loop-in conformation. It may be noted that in EcDHOase intermolecular forces act synergistically with the loop-in conformation in this subunit, as the loop-out conformation would create steric conflicts with neighboring molecules in the crystal (Lee *et al.*, 2005 ▸).

**Figure 4:**
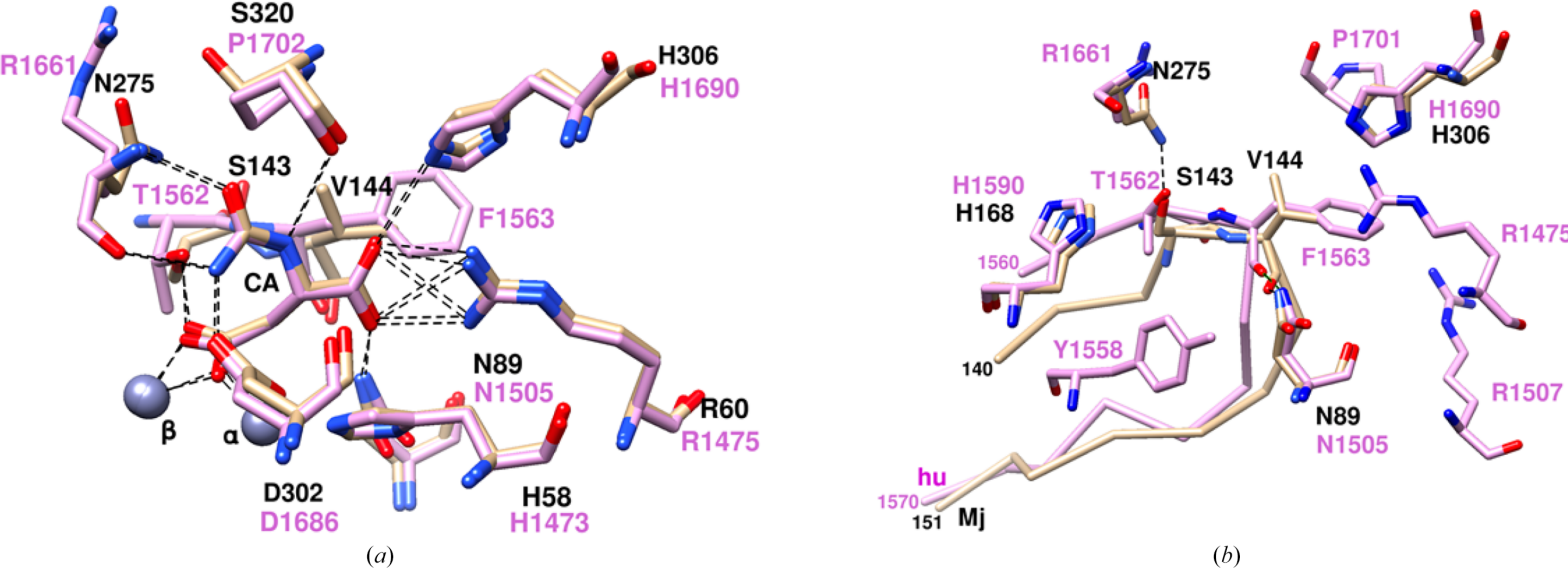
Comparison of the interactions in the active site between MjDHOase and huDHOase (PDB entry 4c6f; Grande-García *et al.*, 2014 ▸). MjDHOase is in tan and huDHOase is in plum. Labels are in black for MjDHOase and plum for huDHOase. (*a*) Only interactions involving CA are shown for clarity. CA is at the center. The figure illustrates the high similarity of the active site in the two proteins. (*b*) Comparison of the interactions of the two residues at the tip of the flexible loop that interact with CA with the protein residues nearby. The figure illustrates the correspondence of some of the interactions and the larger number of interactions in huDHOase compared with MjDHOase (see also Supplementary Table S2). Hydrogen bonds are indicated by dashed lines. Protein residues that make contacts of ≤4.0 Å with these two residues are also shown. The C^α^ backbones of the two flexible loops are included. Ser143 and Val144 in MjDHOase form one hydrogen bond each (Ser143 OG⋯Asn275 ND2 and Asn89 ND2⋯Val144 8O). Ser143 makes contacts with His168, and Val144 makes contacts with His306. Only one corresponding hydrogen bond is formed in huDHOase (Asn1505 ND2⋯Phe1563 O). However, both Thr1562 and Phe1563 form many contacts. Thr1562 makes contacts with Tyr1558, His1590 and Arg166. Phe1563 makes contacts with His1690, Arg1475, Asn1505, Arg1507 and Pro1701. His1690 (huDHOase) and His306 (MjDHOase) are at corresponding positions and His1590 (huDHOase) and His168 (MjDHOase) are at corresponding positions.

Human DHOase (PDB entries 4c6c, 4c6f, 4c6e, 4c6d, 4c6j and 4c6k; Grande-García *et al.*, 2014 ▸) has both CA and DHO in the active site superimposed on each other and both conformations of the flexible loop (residues 1560–1570), loop-in and loop-out, and the occupancy factors of the two ligands and the two conformations were consistent with the loop-in conformation for the CA ligand and the loop-out conformation for the DHO ligand. The loop-in conformation is stabilized by a hydrogen bond from Thr1562 to the β-carboxyl group, while Phe1563 forms stacking interactions with the α-carboxyl of the substrate. In addition, there are several interactions of these two flexible loop residues with the protein, further stabilizing the loop-in conformation. Phe1563 makes many contacts with the side chains of protein residues His1690, Arg1475, Asn1505, Arg1507 and Pro1701, as well as an Asn1505 ND2⋯Phe1563 O hydrogen bond. Thr1562 makes contacts with Tyr1558, His1590 and Arg1661. These interactions are shown in Fig. 4 ▸(*b*) and Supplementary Table S2. As in EcDHOase, the closure of the flexible loop was considered to be essential for activity, and mutations of Phe1563 that precluded closure of the flexible loop exhibited diminished activity (del Caño-Ochoa *et al.*, 2018 ▸). There are no intermolecular interactions with the flexible loop in the crystals of huDHOase and therefore the observations in the crystalline form reflect the situation in solution.

Accordingly, it was unexpected that in MjDHOase the flexible loop (residues 140–151) adopts both loop-in and loop-out conformations in the presence of the CA substrate, with the loop-out conformation having higher occupancy and the loop-in conformation corresponding to weaker electron density. Based on the studies of EcDHOase and huDHOase, we would expect only the loop-in form. These observations suggest that the interactions between the loop-in conformation and CA may not be sufficiently strong under the crystallization conditions to favor the loop-in conformation. While Ser143 provides a hydroxyl for hydrogen bonding to the β-carboxylate group, it was suggested that a Ser residue may be less effective than a Thr residue due to the fact that Ser has more conformational flexibility than Thr (Lee, Chan *et al.*, 2007 ▸; Lee *et al.*, 2007 ▸). In both EcDHOase and huDHOase, the β-carboxylate hydrogen-bonds to a Thr residue. It may be noted that a Thr109Ser mutation in EcDHOase resulted in very weak electron density for the flexible loop, which was not modeled, and reduced the activity of the enzyme (Lee, Chan *et al.*, 2007 ▸). Furthermore, the interaction of Val144 with the α-carboxylate group is not as strong as the hydrogen bond formed to it by a Thr residue, which is the case for Thr110 in EcDHOase. Furthermore, because of its smaller size and aliphatic character, the interaction of Val144 with the α-carboxylate is weaker compared with the aromatic Phe1563 in huDHOase. In addition, the interactions of these two residues at the tip of the flexible loop with the rest of the protein are fewer in MjDHOase compared with EcDHOase and huDHOase (Supplementary Table S2, Figs. 3 ▸*b* and 4 ▸*b*), providing less stability to the loop-in conformation in this system. In contrast, the loop-out conformation involves more hydrogen bonds and more contacts of Ser143 and Val144 with the protein compared with the loop-in conformation (Supplementary Table S3, Fig. 2 ▸*c*), providing a possible explanation for the higher occupancy of the loop-out form, in spite of the presence of CA in the active site. It may be noted that in MjDHOase the observations in the crystalline state should correspond to those for the molecule in solution. The reason is that the flexible loop in MjDHOase is not involved in any intermolecular interactions, except for a salt bridge between Asp151 and Lys378 of a neighboring molecule at (*y*, *x* + 1, −*z*) (Asp151 OD2⋯Lys378′ NZ, 3.76 Å) and this salt bridge is common to both alternate conformations.

Ligands other than CA and DHO interact with the same invariant residues in the active site and adopt one or the other conformation for the flexible loop depending on the functional groups in them and their similarity to the natural substrate and product, the DHOase subtype and organism and, possibly, the crystal preparation. For example, in EcDHOase with the inhibitor 2-oxo-1,2,3,6-tetrahydropyrimidine-4,6-dicarboxylate, which closely resembles both the substrate and product, one subunit adopted the loop-in conformation and the other the loop-out conformation (PDB entry 2eg7; Lee, Chan *et al.*, 2007 ▸). *Yersinia pestis* DHOase with malic acid, which is a close analog of CA, adopts the loop-in conformation (PDB entry 6cty; Lipowska *et al.*, 2019 ▸). HuDHOase adopts the loop-in conformation with both malic acid (PDB entry 8gw0) and 5-fluorouracil (PDB entry 8gvz) (Lin *et al.*, 2023 ▸). ScDHOase with a number of nonsubstrate ligands, including malate (PDB entries 6l0a, 6l0g, 6l0h, 6l0i, 6l0j and 6l0k; Guan *et al.*, 2021*a* ▸,*b* ▸), 5-fluorouracil (PDB entry 6l0b) and 5-aminouracil (PDB entry 6l0f) (Guan *et al.*, 2021*b* ▸), and plumbagin (PDB entry 7ca1; Guan *et al.*, 2021*d* ▸), as well as 5-fluoroorotate (PDB entry 7ca0; Guan *et al.*, 2021*c* ▸), adopted only the loop-in conformation. However, in addition to the different DHOase subtype (type II active DHOase from fungi), these crystals were either grown in the presence of malate (PDB entries 6l0b and 6l0f; Guan *et al.*, 2021*a* ▸,*b* ▸) and/or the ligand was diffused into DHOase–malate crystals (PDB entries 7ca0 and 7ca1; Guan *et al.*, 2021*c* ▸), likely replacing the bound malate in the loop-in conformation. An interesting ligand is 5-fluoroorotate, which is a product mimic, and we would expect the loop-out conformation. This is the case in EcDHOase (PDB entries 2eg8 and 2e25; Lee, Maher & Guss, 2007 ▸) and huDHOase (PDB entries 4c6l, 4c6m, 8pbg, 8pbi, 8pbk, 8pbn and 8pbu; Grande-García *et al.*, 2014 ▸; del Caño-Ochoa *et al.*, 2023 ▸), but ScDHOase adopts the loop-in conformation and a different mode of binding of 5-fluoroorotate (PDB entry 7ca0; Guan *et al.*, 2021*c* ▸), emphasizing the importance of both the different DHOase subtype and the fact that 5-fluoroorotate diffuses into the position of malate in crystals that already have the loop-in flexible loop form during crystal preparation.

## 4. Conclusion

We have determined the structure of MjDHOase grown in the presence of DHO at pH 6.5. During crystallization, the degradative reaction took place and DHO was converted to CA. The active site was similar to EcDHOase and huDHOase with CA bound. However, contrary to expectations, the flexible loop was more in the loop-out conformation, not interacting with the substrate, and to a lesser extent in the loop-in conformation. Additional studies with substrate analogs that resemble CA and different crystallization conditions would provide further insight into the conformation of the flexible loop in this system.

## Supplementary Material

PDB reference: *M. jannaschii* dihydroorotase. complex with carbamoyl aspartate, 9yd7

Supplementary Figures and Tables. DOI: 10.1107/S2053230X25010556/rl5206sup1.pdf

## Figures and Tables

**Table 1:** Data-collection statistics Values in parentheses are for the outer shell.

X-ray source	Beamline 4.2.2, ALS
Wavelength (Å)	1.000
Resolution (Å)	48.23–1.87 (1.91–1.87)
Space group	*P*3_2_21
*a*, *b*, *c* (Å)	111.38, 111.38, 101.22
No. of observations	412722 (18661)
Unique reflections	59804 (3635)
Multiplicity	6.9 (5.1)
〈*I*/σ(*I*)〉	12.1 (0.9)
*R*_merge_ †	
Within *I*+/*I*−	0.106 (1.498)
All *I*+ and *I*−	0.119 (1.635)
*R*_meas_ ‡	
Within *I*+/*I*−	0.124 (1.831)
All *I*+ and *I*−	0.129 (1.812)
*R*_p.i.m._ §	
Within *I*+/*I*−	0.064 (1.036)
All *I*+ and *I*−	0.048 (0.764)
CC_1/2_	0.998 (0.371)
Completeness (%)	99.3 (93.7)
Anomalous multiplicity	3.5 (2.6)
Anomalous completeness (%)	98.8 (91.3)

† The merging *R* factor is defined as *R*_merge_ = 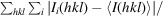

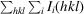
.

‡ *R*_meas_ = 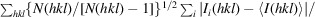

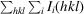
, where *N*(*hkl*) is the number of independent measurements for the reflection with indices *hkl*.

§ *R*_p.i.m._ = 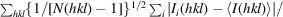

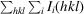
.

**Table 2:** Final refinement statistics Values in parentheses are for the outer shell.

Resolution range (Å)	48.23–1.87 (1.937–1.87)
Reflections used in refinement	59680 (5533)
Reflections used for *R*_free_	2964 (293)
*R*_work_	0.169 (0.286)
*R*_free_	0.186 (0.312)
Wilson *B* factor (Å^2^)	25.79
No. of non H atoms	
Total	3658
Protein†	3449
Ligands (CA)	12
Ions (Zn)	11
Waters	186
Average *B* factors (Å^2^)	
Overall	30.84
Protein	30.47
Ligands (CA)	25.097
Ions (Zn)	34.54
Waters	37.84
Protein residues	423
R.m.s.d., bond lengths (Å)	0.008
R.m.s.d., bond angles (°)	0.96
Ramachandran plot	
Favored (%)	95.93
Outliers (%)	0.96
Rotamer outliers (%)	1.54
Clashscore	3.11

† When computing the number of atoms, an occupancy of 1 was used for the ligand and all zinc ions. KCX is counted as a protein residue.
