# Supplementary material for: Crystal structure of Methanococcus jannaschii dihydroorotase with substrate bound
Source: Acta Crystallogr F Struct Biol Commun. 2026 Jan 1;82(Pt 1):23–31. doi: 10.1107/S2053230X25010556 (PMC12809426; doi:10.1107/S2053230X25010556)
Supplement: Supplementary file 1 [file f-82-00023-sup1.pdf]

Acta Cryst F

STRUCTURAL BIOLOGY
COMMUNICATIONS

Volume 81 (2025)

Supporting information for article:

Crystal structure of *Methanococcus jannaschii* dihydroorotase with substrate bound

Jacqueline Vitali, Jay C. Nix, Haley E. Newman and Michael J. Colaneri

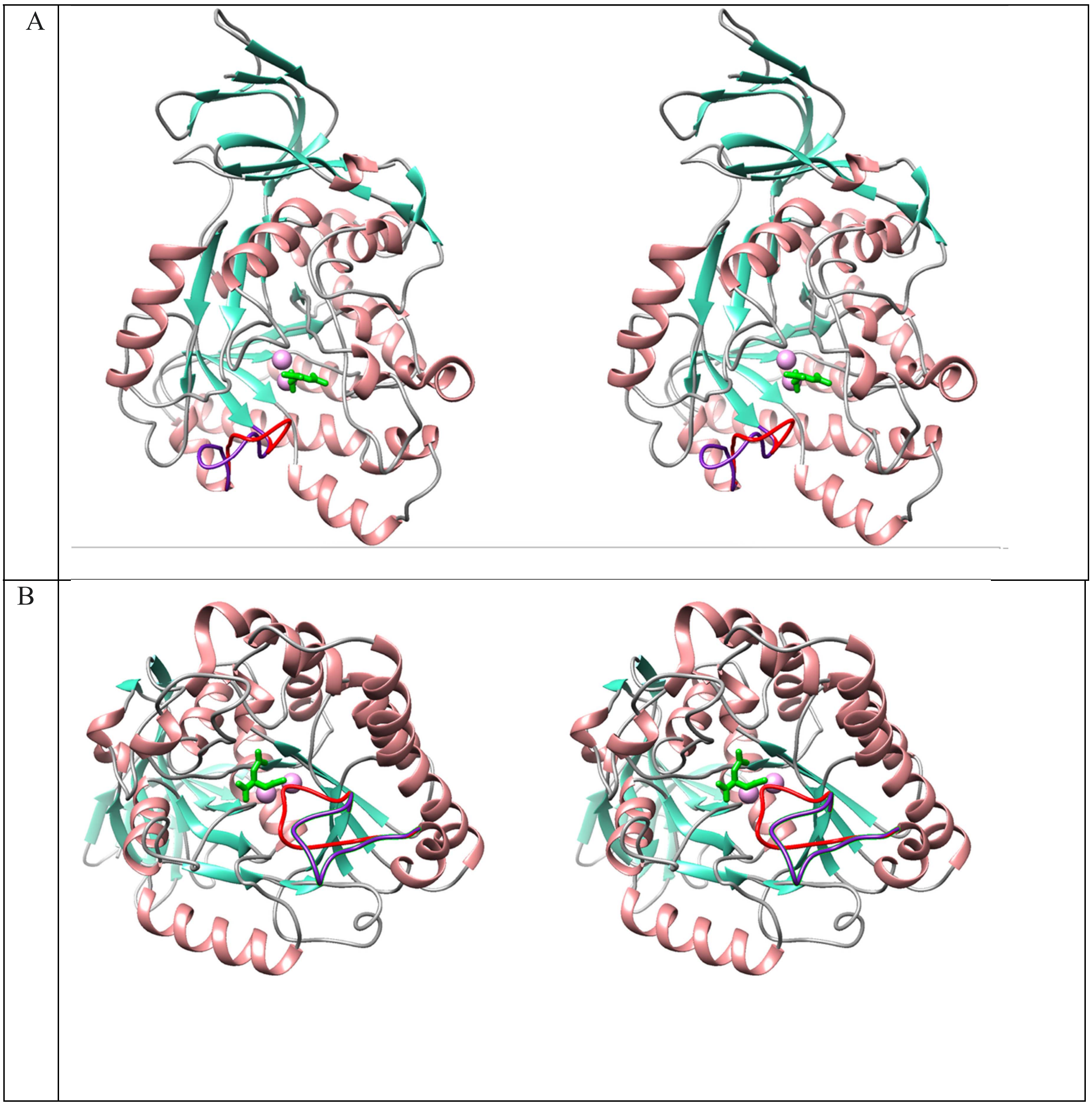

Figure S1 Stereo views of cartoon diagrams of MjDHOase with the flexible loop in the two alternate conformations. The two diagrams are approximately perpendicular to each other. Helices are depicted in salmon color, strands in teal and loops in gray except for the flexible loop that is shown in red for the loop-in conformation and purple for the loop-out conformation. The two Zn ions are shown as spheres of plum color and the substrate CA in the active site is in lime-green color.

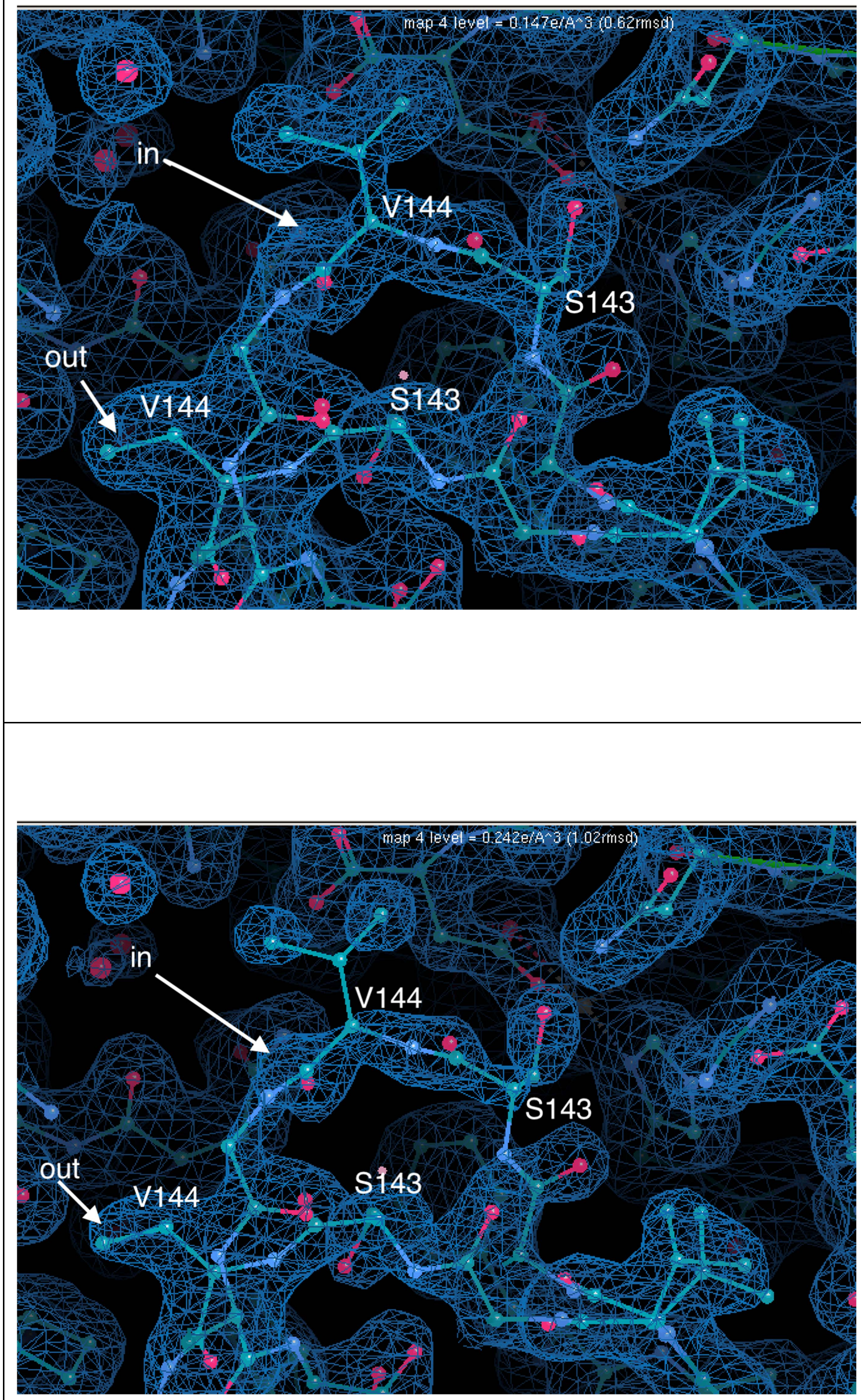

Figure S2 The 2mFo-DFc map at two different contour levels for the loop-in and loop-out conformations at the positions of Ser143 and V144. The figure was prepared using coot.

Table S1 Hydrogen bonds, salt bridges and coordinate bonds of CA with DHOase. All distances are in Å.

**Salt bridges**		
ARG60 NH1	CA424 O61	2.80
ARG60 NH2	CA424 O61	3.52
ARG60 NH1	CA424 O62	3.55
ARG60 NH2	CA424 O62	2.79
**Hydrogen bonds**		
ASN89 ND2	CA424 O61	3.04
HIS306 NE2	CA424 O62	3.26
CA424 N1	SER320 O	3.32
CA424 N3	ASN275 O	2.84
CA424 N3	ASP302 OD2	2.71
ASN275 N	CA424 O2	2.73
SER143 OG (loop-in)	CA424 O5	2.63
**Coordinate bonds**		
ZN501	CA424 O4	1.98
ZN502	CA424 O4	2.38
ZN502	CA424 O5	2.43

Table S2 Hydrogen bonds and contacts (≤ 4 Å) between the two residues on the tip of the flexible loop that interact with CA in the loop-in conformation and the surrounding protein.

The number of contacts between two residues is indicated in parentheses. Three dots (…) indicate hydrogen bonds, three dashes (---) indicate contacts between two residues. Corresponding interactions in the three proteins are shown in rows. The corresponding hydrogen bonds involve invariant protein residues. The hydrogen bond with the side chain of invariant position N275 in MjDHOase is replaced with contacts with the side chains of corresponding residues L222 in EcDHOase and R1661 in huDHOase. One letter code for amino acids is used in this table. For hydrogen bonds, the donor atom is on the left of the interaction.

MjDHOase		EcDHOase (1XGE.pdb)		HuDHOase (4C6F.pdb)	
Hydrogen Bonds (Å)					
N275 ND2…S143 OG	3.04				
N89 ND2 … V144 O	2.74	N44 ND2 … T110 O	3.13	N1505 ND2 … F1563 O	2.70
		T110 OG1 … A266 O	3.41		
		T109 N … S112 OG	2.92		
Contacts (≤ 4 Å)					
S143 --- H168 (2)		T109 --- H139 (2)		T1562 --- H1590 (5)	
		T109 --- L222 (3)		T1562 --- R1661 (2)	
		T109 --- Y104 (2)		T1562 --- Y1558 (1)	
V144 --- H306 (1)				F1563 --- H1690 (6)	
				F1563 --- R1475 (2)	
				F1563 --- N1505 (1)	
				F1563 --- R1507 (1)	
				F1563 --- P1701 (1)	

Table S3 Hydrogen bonds and contacts (≤ 4 Å) between S143 and V144 of the flexible loop in both loop-in and loop-out conformations with the surrounding protein

The number of contacts between two residues is indicated in parentheses. Three dots (…) indicate hydrogen bonds, three dashes (---) indicate contacts between two residues. One letter code for amino acids is used in this table. For hydrogen bonds, the donor atom is on the left of the interaction.

MjDHOase loop-in		MjDHOase loop-out	
Hydrogen Bonds (Å)			
N275 ND2…S143 OG	3.04	S143 OG…L147 O	2.47
		S143 N…L147 O	3.58
N89 ND2 … V144 O	2.74	L147 N… V144 O	3.03
Contacts (≤ 4 Å)			
S143 --- H168 (2)		S143 --- F139 (5)	
		S143 --- L147 (3)	
V144 --- H306 (1)		V144 --- N89 (1)	
		V144 --- K91 (1)	
		V144 --- P93 (2)	
